# Correction: Pathways to sustainable lithium-mediated ammonia electrosynthesis: tuning reaction environment and advancing reactor design

**DOI:** 10.1039/d6ra90081j

**Published:** 2026-07-27

**Authors:** Ngoc-Trung Nguyen, Thi Kim Cuong Phu, Minh Triet Nguyen, Trang Minh Pham, Phi Long Nguyen, Thi Viet Bac Phung

**Affiliations:** a Center for Environmental Intelligence, VinUniversity Hanoi Vietnam bac.ptv@vinuni.edu.vn; b School of Electrical and Electronic Engineering, Hanoi University of Industry Hanoi Vietnam; c College of Engineering and Computer Science, VinUniversity Hanoi Vietnam

## Abstract

Correction for ‘Pathways to sustainable lithium-mediated ammonia electrosynthesis: tuning reaction environment and advancing reactor design’ by Ngoc-Trung Nguyen *et al.*, *RSC Adv.*, 2026, https://doi.org/10.1039/d6ra02156e.

The authors regret the incorrect affiliations listed on the original manuscript, where the institutions listed for b and c were the wrong way round. The corrected affiliations for this paper are as shown here.

The Royal Society of Chemistry apologises for these errors and any consequent inconvenience to authors and readers.

